# Supplementary material for: Role and mechanism of NCAPD3 in promoting malignant behaviors in gastric cancer
Source: Front Pharmacol. 2024 Apr 22;15:1341039. doi: 10.3389/fphar.2024.1341039 (PMC11070777; doi:10.3389/fphar.2024.1341039)
Supplement: Supplementary file 11 [file DataSheet2.ZIP › GSEA/Transcipt factor/my_analysis.Gsea.1599461814734/ATF1_Q6.html]

Details for gene set ATF1\_Q6[GSEA]

|  || Dataset | filtered\_dataset.sample\_info.cls#WT\_versus\_NCAPD3\_MUT |
| Phenotype | sample\_info.cls#WT\_versus\_NCAPD3\_MUT |
| Upregulated in class | NCAPD3\_MUT |
| GeneSet | ATF1\_Q6 |
| Enrichment Score (ES) | -0.4345472 |
| Normalized Enrichment Score (NES) | -2.0741835 |
| Nominal p-value | 0.0 |
| FDR q-value | 0.04936091 |
| FWER p-Value | 0.398 |
Table: GSEA Results Summary

  

Fig 1: Enrichment plot: ATF1\_Q6      
 Profile of the Running ES Score & Positions of GeneSet Members on the Rank Ordered List

  

| SYMBOL | TITLE | RANK IN GENE LIST | RANK METRIC SCORE | RUNNING ES | CORE ENRICHMENT || 1 | 7763 | ZFAND5 | 26 | 1.006 | 0.0625 | No |
| 2 | 4091 | SMAD6 | 519 | 0.462 | -0.2544 | No |
| 3 | 23235 | SIK2 | 523 | 0.460 | -0.2195 | No |
| 4 | 5198 | PFAS | 570 | 0.437 | -0.2173 | No |
| 5 | 1973 | EIF4A1 | 804 | 0.319 | -0.3593 | No |
| 6 | 8904 | CPNE1 | 814 | 0.308 | -0.3410 | No |
| 7 | 384 | ARG2 | 945 | -0.366 | -0.4050 | Yes |
| 8 | 7067 | THRA | 976 | -0.392 | -0.3949 | Yes |
| 9 | 687 | KLF9 | 996 | -0.402 | -0.3762 | Yes |
| 10 | 10938 | EHD1 | 1019 | -0.419 | -0.3582 | Yes |
| 11 | 23764 | MAFF | 1039 | -0.436 | -0.3367 | Yes |
| 12 | 8444 | DYRK3 | 1131 | -0.502 | -0.3617 | Yes |
| 13 | 1540 | CYLD | 1162 | -0.530 | -0.3405 | Yes |
| 14 | 665 | BNIP3L | 1185 | -0.556 | -0.3115 | Yes |
| 15 | 5154 | PDGFA | 1191 | -0.566 | -0.2695 | Yes |
| 16 | 10150 | MBNL2 | 1217 | -0.591 | -0.2398 | Yes |
| 17 | 10221 | TRIB1 | 1229 | -0.604 | -0.1990 | Yes |
| 18 | 5187 | PER1 | 1254 | -0.641 | -0.1645 | Yes |
| 19 | 1649 | DDIT3 | 1341 | -0.779 | -0.1635 | Yes |
| 20 | 219539 | YPEL4 | 1354 | -0.803 | -0.1074 | Yes |
| 21 | 22936 | ELL2 | 1370 | -0.882 | -0.0470 | Yes |
| 22 | 64764 | CREB3L2 | 1382 | -0.930 | 0.0202 | Yes |
Table: GSEA details [plain text format]

  

Fig 2: ATF1\_Q6      
 Blue-Pink O' Gram in the Space of the Analyzed GeneSet

  

Fig 3: ATF1\_Q6: Random ES distribution      
 Gene set null distribution of ES for **ATF1\_Q6**
